# Depressive symptoms and objectively measured physical activity and sedentary behaviour throughout adolescence: a prospective cohort study

**DOI:** 10.1016/S2215-0366(20)30034-1

**Published:** 2020-03

**Authors:** Aaron Kandola, Gemma Lewis, David P J Osborn, Brendon Stubbs, Joseph F Hayes

**Affiliations:** a Division of Psychiatry, University College London, London, UK; b Camden and Islington National Health Service Foundation Trust, London, UK; c Department of Psychological Medicine, Institute of Psychiatry, Psychology, and Neuroscience, King's College London, London, UK; d Physiotherapy Department, South London and Maudsley National Health Service Foundation Trust, London, UK

## Abstract

**Background:**

Identifying modifiable risk factors is essential to reduce the prevalence adolescent depression. Self-report data suggest that physical activity and sedentary behaviour might be associated with depressive symptoms in adolescents. We examined associations between depressive symptoms and objectively measured physical activity and sedentary behaviour in adolescents.

**Methods:**

From a population-based cohort of adolescents whose mothers were invited to participate in the Avon Longitudinal Study of Parents and Children (ALSPAC) study, we included participants with at least one accelerometer recording and a Clinical Interview Schedule-Revised (CIS-R) depression score at age 17·8 years (reported as age 18 years hereafter). Amounts of time spent in sedentary behaviour and physical activity (light or moderate-to-vigorous) were measured with accelerometers at around 12 years, 14 years, and 16 years of age. Total physical activity was also recorded as count per minute (CPM), with raw accelerometer counts averaged over 60 s epochs. Associations between the physical activity and sedentary behaviour variables and depression (CIS-R) scores at age 18 years were analysed with regression and group-based trajectory modelling.

**Findings:**

4257 adolescents from the 14 901 enrolled in the ALSPAC study had a CIS-R depression score at age 18 years. Longitudinal analyses included 2486 participants at age 12 years, 1938 at age 14 years, and 1220 at age 16 years. Total follow-up time was 6 years. Total physical activity decreased between 12 years and 16 years of age, driven by decreasing durations of light activity (mean 325·66 min/day [SD 58·09] at 12 years; 244·94 min/day [55·08] at 16 years) and increasing sedentary behaviour (430·99 min/day [65·80]; 523·02 min/day [65·25]). Higher depression scores at 18 years were associated with a 60 min/day increase in sedentary behaviour at 12 years (incidence rate ratio [IRR] 1·111 [95% CI 1·051–1·176]), 14 years (1·080 [1·012–1·152]), and 16 years of age (1·107 [1·015–1·208]). Depression scores at 18 years were lower for every additional 60 min/day of light activity at 12 years (0·904 [0·850–0·961]), 14 years (0·922 [0·857–0·992]), and 16 years of age (0·889 [0·809–0·974]). Group-based trajectory modelling across 12–16 years of age identified three latent subgroups of sedentary behaviour and activity levels. Depression scores were higher in those with persistently high (IRR 1·282 [95% CI 1·061–1·548]) and persistently average (1·249 [1·078–1·446]) sedentary behaviour compared with those with persistently low sedentary behaviour, and were lower in those with persistently high levels of light activity (0·804 [0·652–0·990]) compared with those with persistently low levels of light activity. Moderate-to-vigorous physical activity (per 15 min/day increase) at age 12 years (0·910 [0·857–0·966]) and total physical activity (per 100 CPM increase) at ages 12 years (0·941 [0·910–0·972]) and 14 years (0·965 [0·932–0·999]), were negatively associated with depressive symptoms.

**Interpretation:**

Sedentary behaviour displaces light activity throughout adolescence, and is associated with a greater risk of depressive symptoms at 18 years of age. Increasing light activity and decreasing sedentary behaviour during adolescence could be an important target for public health interventions aimed at reducing the prevalence of depression.

**Funding:**

Details of funding are provided in the Acknowledgments.

## Introduction

Depression affects around 300 million people worldwide.^1^ Among adolescents, the prevalence of depression appears to be increasing, with one US-based study showing an increase from about 8·7% in 2005 to 11·3% in 2014.^2^ The first onset of depression tends to be during adolescence, which represents an important window for identifying modifiable risk factors and intervening to prevent depression in later life.^3^, ^4^ Increasing evidence suggests that physical activity can reduce the symptoms of depression in clinical and non-clinical populations. Most previous studies have focused on physical activity of moderate-to-vigorous intensity, such as brisk walking or cycling, which appear to modulate several biological and psychosocial pathways to reduce depressive symptoms.^5^ However, evidence also suggests that time spent in sedentary behaviour is associated with the risk of depression in adults.^6^, ^7^

**Research in context**

**Evidence before this study**

Identifying modifiable risk factors for adolescent depression is a global priority. Low physical activity and high sedentary behaviour could be important early risk factors for depression that are modifiable through practical population-level approaches with relatively few risks. We conducted a systematic literature search in PubMed up to Oct 23, 2019, using the search terms “physical activity OR exercise OR accelerometer AND adolescen* AND depress* OR anxiety”. We also manually searched the reference lists of relevant review papers. We found some evidence from population-based studies that total or moderate-to-vigorous physical activity levels were negatively associated with the risk of depression. Sedentary behaviour was positively associated with depression risk. Most previous research has been in adults, but the first onset of depression tends to be during adolescence and findings in this age group have been inconsistent. Nearly all previous studies have used self-report measures of activity, which are prone to bias, provide unreliable estimates, and do not sufficiently account for sedentary behaviour or light activity, which comprise most of daily waking activity. The few studies in the field of mental health that have used devices to objectively measure activity have mostly been cross-sectional studies or studies lacking repeated measures to examine changes over time. The single prospective study identified in our search that used objective physical activity measures in adolescents found no association between total or moderate-to-vigorous activity levels and depression. However, the study was limited by its small sample size, did not record sedentary behaviour or light activity, included only one measure of physical activity (at baseline), and was unable to examine the effect of activity changes over time during adolescence. Several studies have used objective measures to show that sedentary behaviour progressively displaces light activity throughout adolescence, but the implications of this activity shift—including the potential harms of sedentary behaviour and benefits of light activity—are unknown, and more high-quality evidence is needed.

**Added value of this study**

This is the first prospective cohort study to objectively measure physical activity and sedentary behaviour using repeated assessments, and to examine their associations with depressive symptoms in a large, population-based sample of adolescents. Our study provides high-quality evidence to validate widespread concerns over the potential dangers of globally rising sedentary behaviour in young people, by showing consistent associations with depressive symptoms. While nearly all previous studies have only focused on total and moderate-to-vigorous physical activity, our findings suggest that light activity might have benefits for reducing the risk of depressive symptoms.

**Implications of all the available evidence**

Global action is needed to reduce the burden of adolescent depression. Our findings highlight the displacement of light activity with sedentary behaviour during adolescence as a potentially important target for public health interventions to reduce the risk of depression. Public health guidelines and interventions aimed at increasing activity in young people have focused mainly on its physical health benefits, such as improving coordination skills or bone strength, but such developmental or physical health complications are not common. Emphasising the mental health benefits of activity would send a stronger and more widely relatable message given the rising prevalence of depression in adolescents. Our findings suggest that even a 2 h reduction in daily sedentary behaviour could substantially reduce the risk of depressive symptoms in adolescents. As sedentary behaviour increases during adolescence, guidelines and interventions need to include measurable and achievable targets for reducing sedentary behaviour in young people. The importance of light activity, which could reduce the risk of depressive symptoms, should also be highlighted. Light activity requires less effort than moderate or vigorous activity, does not require planned or dedicated time periods, and can be easily and simply incorporated into a daily routine, such as standing or active classes. As a result, light activity might yield a more sustained uptake and be easier to implement at a population level than moderate-to-vigorous activity.

Findings in adolescents have been scarce and inconsistent with regard to the associations between depressive symptoms and overall physical activity, moderate-to-vigorous physical activity,^8^, ^9^, ^10^, ^11^, ^12^, ^13^ or sedentary behaviour.^14^, ^15^, ^16^ Nearly all previous studies in adolescents used self-report physical activity measures, which are subject to mood, attention, recall, and social desirability biases; obtaining reliable estimates of physical activity with these self-report measures is challenging in both adults and adolescents.^17^, ^18^ These methods also underestimate sedentary behaviour and rarely account for light-intensity activities.^19^, ^20^ Light activity (eg, walking at a casual pace) has the lowest test–retest reliability from self-report measures of all physical activity intensities, possibly because of its unstructured nature and dispersion throughout the day.^21^ Consequently, light activity has been under-studied compared with moderate or vigorous activity. However, light activity and sedentary behaviour constitute the vast majority of waking daily activity, and better methods are therefore necessary to estimate their effects.

Objective measures, such as accelerometers, are rarely used in mental health research, but can provide reliable estimates of total physical activity and time spent in sedentary behaviour, light activity, and moderate-to-vigorous physical activity.^22^ To the best of our knowledge, only one prospective study^13^ has examined associations between physical activity and depressive symptoms in adolescents using objective measures with heart-rate and movement sensors, finding no evidence of longitudinal associations between total physical activity or moderate-to-vigorous physical activity at age 15 years and depression at age 17·5 years. However, as the study focused on moderate-to-vigorous physical activity, it might have overlooked associations between depressive symptoms and sedentary behaviour or light activity. Furthermore, by measuring physical activity only at baseline, associations between changes in physical activity over time and depressive symptoms could not be examined.^13^

Total physical activity levels, in terms of self-reported active minutes or energy expenditure per day, decrease by around 7% each year between the ages of 10 years and 19 years.^23^ Accelerometry data suggest that this decrease might be driven by increasing sedentary behaviour and decreasing light activity throughout adolescence,^24^, ^25^, ^26^ but the effects of this activity shift on mental health are unclear. Although there are growing concerns over increasing sedentary behaviours (such as screen time) in young people,^14^ high-quality evidence on whether such behaviours increase the risk of adverse physical health^27^, ^28^ or mental health outcomes, including depression, is lacking.^14^, ^15^, ^16^ In adults, cross-sectional accelerometry data suggest that depressive symptoms are positively associated with sedentary behaviour and negatively associated with light activity.^29^, ^30^, ^31^ Such associations might also exist in adolescents.

To address these key evidence gaps, we examined associations between total physical activity and time spent engaging in sedentary behaviour, light activity, and moderate-to-vigorous physical activity—measured objectively with accelerometers at three points during adolescence—and depressive symptoms at 18 years of age. Our repeated measures design also allowed us to examine how changes in physical activity and sedentary behaviour over time were associated with subsequent depressive symptoms. We hypothesised that total physical activity and time spent engaging in light and moderate-to-vigorous physical activity would be negatively associated with depressive symptoms, and that time spent in sedentary behaviour would be positively associated with depressive symptoms.

## Methods

### Sample

Data for this study were from the Avon Longitudinal Study of Parents and Children (ALSPAC) cohort. The study website contains details of all ALSPAC cohort data, which are available through an online data dictionary and variable search tool. Pregnant women living in the Avon area (South-West England) with an expected delivery date between April 1, 1991, and Dec 31, 1991, were invited to join the ALSPAC study.^32^, ^33^ Data from children who were born to these mothers between these dates and who were alive at 12 months of age were used in our study. To bolster the initial sample, additional recruitment from among the eligible individuals who did not join the ALSPAC study originally was done when the oldest children in the study were around 7 years of age. All participants were followed up regularly through ongoing questionnaires and clinical assessments.

Ethical approval for this study was obtained from the ALSPAC Law and Ethics committee and the local research ethics committees. Informed consent for the use of data collected via questionnaires and clinics was obtained from participants, in accordance with the recommendations of the ALSPAC Ethics and Law Committee at the time. Our sample included participants with at least one accelerometer recording and a Clinical Interview Schedule-Revised (CIS-R) depression score at age 17·8 years (reported as age 18 years for simplicity). A flowchart of ALSPAC participants for this study can be found in the appendix (p 1).

### Measures of physical activity

Physical activity was measured at ages 11·8 years (reported as 12 years), 13·9 years (reported as 14 years), and 15·5 years (reported as 16 years) using accelerometers, the details of which are described elsewhere.^34^, ^35^ Physical activity data were collected with MTI Actigraph 7164 or 71256 accelerometers (Actigraph LLC, Fort Walton Beach, FL, USA) worn on the right hip for 7 days. Both are of the same generation of uniaxial accelerometers and there are no significant differences between their outputs in comparison studies.^36^ Actigraph accelerometers have been validated for use in young people against indirect calorimetry.^22^

Participants wore accelerometers during waking hours, except when washing or doing water sports. Data were recorded in raw accelerometer counts and averaged over 60 s epochs to create a count per minute (CPM) variable. We only included data from participants who recorded more than 10 h of wear time for at least 3 days, as previously shown to provide good statistical power and reliability.^35^

We calculated total physical activity as the average CPM per day, from the full recording period. This measure accounts for the amount and intensity of physical activity undertaken and its use is validated against the doubly labelled water method.^37^ Time spent at different physical activity intensities was calculated as the mean number of minutes spent in predefined intensity categories from a calibration study in a subsample of 246 children from the ALSPAC study.^35^ Three intensity categories were defined: moderate-to-vigorous physical activity (≥3600 CPM; eg, brisk walking or jogging), light activity (200–3599 CPM; eg, slow walking), and sedentary behaviour (≤199 CPM; eg, lying or sitting still). We also calculated the time spent in each activity intensity as a percentage of total wear time to account for differences in wear time.

### Outcomes

Our primary outcome was the presence of depressive symptoms, measured as a computerised CIS-R depression score completed by participants at assessment clinics at around 18 years of age. The CIS-R is commonly used for assessing depression and anxiety according to ICD-10 criteria.^38^ It gives a score for the presence and severity of depressive symptoms in the past week, ranging from 0 to 21.

We used the short Moods and Feelings Questionnaire (MFQ) to assess baseline depression at age 12 years, as well as at ages 14 years and 16 years. The MFQ is a self-report measure of symptoms over the past 2 weeks, with scores ranging from 0 to 26. It is validated for assessing depressive symptoms in adolescents in population-based research.^39^

As depressive symptoms exist on a continuum, we use continuous depression scores as our primary outcome in the main analyses. For other analyses, we defined possible cases of depression as scores of at least 10 on the MFQ^40^ and at least 7 on the CIS-R.^41^

### Covariates

We included several covariates in our models, based on existing literature in the field and the use of directed acyclic graphs (DAGs; appendix p 2). The covariates in our main analyses included sex, ethnicity, maternal social class (manual or non-manual employment, based on the UK Registrar General's occupational coding system), baseline depression (MFQ), IQ (measured at age 8 years), parental psychiatric history (diagnoses of depression or schizophrenia), parental education (secondary level or degree-level or higher education), and total accelerometer wear time.

As alcohol use and smoking were measured after baseline, at 15 years (for smoking) and 16 years of age (for alcohol use), they were only included as covariates in sensitivity analyses. Our DAG indicated body-mass index (BMI) as an intermediate variable on the causal pathway between physical activity or sedentary behaviour and depression. To avoid over-adjustment,^42^ we include BMI in sensitivity analyses.

### Data analysis

We calculated means and SDs for normally distributed, continuous variables, and medians and IQRs for those with a non-normal distribution.

To assess cross-sectional associations between physical activity, sedentary behaviour, and depressive symptoms (MFQ) at baseline (age 12 years), we included total physical activity (mean CPM) and time spent in sedentary behaviour, light activity, and moderate-to-vigorous activity as exposure variables in separate negative binomial regression models. We chose this model because the MFQ and CIS-R outcome variables were highly positively skewed due to over-dispersion (appendix p 3). In line with previous studies, we used units of 15 min for moderate-to-vigorous activity, 60 min for light activity and sedentary behaviour, and 100 CPM for total activity, to avoid large numbers of minutes and counts producing very small model coefficients that are hard to interpret. We first ran univariate models, before fully adjusted models. In models with total activity as the exposure variable, we did not adjust for total wear time because total CPM already accounts for time.

We also investigated the longitudinal associations between physical activity and sedentary behaviour at ages 12 years, 14 years, and 16 years with depressive symptoms at age 18 years, measured with CIS-R. We used the same approach as in the cross-sectional models, but our outcome variable was CIS-R depression score. We ran separate models for each timepoint to avoid collinearity from including multiple timepoints in the same model.

The associations between different physical activity and sedentary behaviour trajectories over time and depressive symptoms were investigated to better account for the time-varying nature of physical activity, sedentary behaviour, and depressive symptoms. We used group-based trajectory modelling^43^ to identify unobserved (latent) subgroups of participants with statistically similar trajectories of total physical activity, sedentary behaviour, light activity, and moderate-to-vigorous physical activity time through ages 12 years, 14 years, and 16 years (appendix p 1).

The associations between physical activity trajectory groups and depressive symptoms at age 18 years were assessed with negative binomial regression with the same adjustments as in our main analysis. We used group-based trajectory modelling to generate trajectories of depressive symptoms through ages 12 years, 14 years, and 16 years using MFQ scores. We then entered this as a categorical covariate into the regression models, instead of baseline depression, to adjust for varying depressive symptoms throughout adolescence.

In sensitivity analyses, determined a priori, we re-ran our main analyses with several different modifications: excluding participants with elevated depressive symptoms at baseline (MFQ score ≥10), to minimise the risk of reverse causation with depressive symptoms explaining physical activity and sedentary behaviour levels; with BMI as a confounder; with smoking and alcohol use as confounders; with sex as an effect modifier (because there were sex differences in physical activity levels); and with depression as a binary outcome using logistic regression models.

To reduce the risk of bias from missing data, we conducted multiple imputation using chained equations for all participants with a CIS-R score (n=4257), generating 30 imputed datasets. We re-ran our main analysis in this imputed sample and compared the results with our non-imputed sample (see appendix p 12 for full details).

All analyses were done with Stata version 16.

### Role of the funding source

The funders of the study had no role in study design, data collection, data analysis, data interpretation, or writing of the report. The corresponding author had full access to all the data and had final responsibility for the decision to submit for publication.

## Results

15 454 pregnant women and 14 901 children alive at 12 months of age were enrolled into the ALSPAC study and were followed up regularly. From our sample of 4257 participants with a CIS-R depression score at age 18 years, the complete-case cross-sectional analysis included 3352 participants, and the longitudinal analyses included 2486 participants at age 12 years, 1938 at age 14 years, and 1220 at age 16 years. Total follow-up time was 6 years.

Table 1 shows the baseline characteristics of participants included in our analysis. A comparison of baseline characteristics of participants with those not included (n=10 664) is shown in the appendix (pp 5–6).

**Table 1:** Baseline characteristics of included participants

	**Participants (n=4257)**
**Sex**	
Male	1867/4257 (43·9%)
Female	2390/4257 (56·1%)
**Ethnicity**	
White	3889/4062 (95·7%)
Other	173/4062 (4·3%)
**Parental education**	
Degree or higher	1207/4257 (28·4%)
Secondary	3050/4257 (71·6%)
**Maternal social class**	
Manual	553/3626 (15·3%)
Non-manual	3073/3626 (84·7%)
**Parental psychiatric diagnosis**	
Severe depression or schizophrenia	422/4257 (9·9%)
No diagnosis	3835/4257 (90·1%)
**Body-mass index**	
≥21 kg/m^2^ (overweight or obese)	270/3671 (7·4%)
<21 kg/m^2^ (normal weight or underweight)	3401/3671 (92·6%)
**Baseline depression**	
Yes (MFQ score ≥10)	344/3683 (9·3%)
No (MFQ score <10)	3339/3683 (90·7%)

Data are n/N (%) where N is the number of participants with non-missing data. MFQ=Moods and Feelings Questionnaire.

Total physical activity levels were higher in boys than in girls at all ages, but the trends across time remained the same (appendix p 6). Mean total daily physical activity decreased from 603·32 CPM (SD 177·62) at age 12 years to 474·83 CPM (158·68) at age 16 years (p<0·0001). Mean duration of sedentary behaviour increased from 430·99 min/day (65·80) at age 12 years to 523·02 min/day (65·25) at age 16 years (p<0·0001). Mean time spent doing light activity decreased from 325·66 min/day (58·09) at age 12 years to 244·94 min/day (55·08) at age 16 years (p<0·0001). Time spent doing moderate-to-vigorous physical activity remained stable over time (figure).

**Figure:**
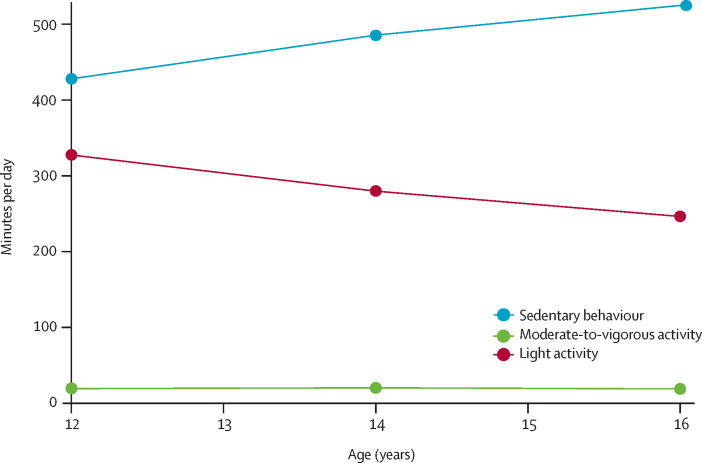
Physical activity levels at different ages

From our sample the median CIS-R depression score at age 18 years was 2 (IQR 0–5) and there were 747 (17·5%) possible cases of depression.

The fully adjusted cross-sectional models indicated that higher total physical activity, time in sedentary behaviour, and time doing light activity were associated with baseline depression (MFQ score), whereas time spent doing moderate-to-vigorous physical activity was not (table 2).

**Table 2:** Cross-sectional associations between baseline depression scores at 12 years and different levels of physical activity and sedentary behaviour at 12 years of age

	**Unadjusted model (n=3352)**		Fully adjusted model*(n=3352)	
	IRR (95% CI)	p value	IRR (95% CI)	p value
Count per minute (per 100)	0·963 (0·947–0·980)	<0·0001	0·981 (0·963–0·100)	0·0454
Sedentary behaviour (per 60 min)	1·049 (1·020–1·080)	0·0014	1·047 (1·014–1·081)	0·0052
Light activity (per 60 min)	0·930 (0·904–0·965)	<0·0001	0·949 (0·916–0·983)	0·0040
Moderate-to-vigorous activity (per 15 mins)	0·955 (0·927–0·984)	0·0031	0·991 (0·959–1·023)	0·5862

Baseline depression was assessed with the Moods and Feelings Questionnaire. IRR=incidence rate ratio.

* Adjusted for sex, maternal social class, parental psychiatric history, parental education, ethnicity, baseline depression, and total accelerometer wear time at each timepoint.

In the fully adjusted longitudinal analysis (table 3), higher total physical activity (CPM) at ages 12 years and 14 years, but not at age 16 years, was associated with a lower depression score at age 18 years. At all three timepoints, a 60 min/day increase in sedentary behaviour was associated with a higher depression score at 18 years of age: 11·1% increase for sedentary behaviour at 12 years, 8·0% at 14 years, and 10·7% at 16 years of age. At all timepoints, each 60 min/day increase in light activity was associated with a lower depression score at 18 years of age: 9·6% for light activity at 12 years, 7·8% at 14 years, and 11·1% at 16 years of age. For every 15 min increase of moderate-to-vigorous physical activity at age 12 years, depression scores were 9·0% lower at age 18 years, but this association was not found for moderate-to-vigorous physical activity at ages 14 years or 16 years (table 3). For all exposures, on the basis of p values, the strength of association with the primary outcome was statistically strongest at age 12 years and was decreased at subsequent timepoints (table 3).

**Table 3:** Longitudinal associations between depression scores at 18 years and different levels of physical activity and sedentary behaviour at 12 years, 14 years, and 16 years of age

	**Unadjusted model**		Fully adjusted model*	
	IRR (95% CI)	p value	IRR (95% CI)	p value
**Exposure at 12 years (n=2486)**				
Count per minute (per 100)	0·910 (0·882–0·939)	<0·0001	0·941 (0·910–0·972)	<0·0001
Sedentary behaviour (per 60 min)	1·108 (1·054–1·165)	<0·0001	1·111 (1·051–1·176)	<0·0001
Light activity (per 60 min)	0·883 (0·834–0·933)	<0·0001	0·904 (0·850–0·961)	0·0012
Moderate-to-vigorous activity (per 15 mins)	0·848 (0·863–0·965)	<0·0001	0·910 (0·857–0·966)	0·0018
**Exposure at 14 years (n=1938)**				
Count per minute (per 100)	0·933 (0·902–0·965)	<0·0001	0·965 (0·932–0·999)	0·0443
Sedentary behaviour (per 60 min)	1·114 (1·057–1·175)	<0·0001	1·080 (1·012–1·152)	0·0200
Light activity (per 60 min)	0·908 (0·851–0·970)	0·0044	0·922 (0·857–0·992)	0·0299
Moderate-to-vigorous activity (per 15 mins)	0·913 (0·863–0·965)	0·0409	0·960 (0·905–1·018)	0·1691
**Exposure at 16 years (n=1220)**				
Count per minute (per 100)	0·939 (0·896–0·983)	0·0072	0·984 (0·937–1·033)	0·5092
Sedentary behaviour (per 60 min)	1·101 (1·026–1·180)	0·0068	1·107 (1·015–1·208)	0·0210
Light activity (per 60 min)	0·882 (0·810–0·961)	0·0040	0·889 (0·809–0·974)	0·0133
Moderate-to-vigorous activity (per 15 mins)	0·938 (0·883–0·997)	0·0413	1·001 (0·936–1·071)	0·9662

Depression at 18 years of age was assessed with the Clinical Interview Schedule-Revised. IRR=incidence rate ratio.

* Adjusted for sex, maternal social class, parental psychiatric history, parental education, ethnicity, baseline depression, and total accelerometer wear time at each timepoint.

We identified two latent subgroups of participants with similar trajectories for total physical activity and three subgroups for each physical activity intensity (table 4). Using the persistently low subgroup as the reference group, we found no association between the trajectories of total physical activity and depressive symptoms. However, compared with those with persistently low levels of sedentary behaviour, depression scores were 24·9% higher in those with persistently average and 28·2% higher in those with persistently high levels of sedentary behaviour. Lower depression scores were identified in participants with persistently high levels of light activity (19·6% lower) and moderate-to-vigorous activity (31·1% lower), compared with those with persistently low levels. However, persistently average levels of light or moderate-to-vigorous physical activity were not associated with depression scores at age 18 years. Graphical representations of each trajectory model in our analyses, as well as model fit indices from all models that we considered, are presented in the appendix (pp 3–5, 6–7).

**Table 4:** Associations of physical activity and sedentary behaviour trajectories with depression scores (n=2486)

	**n (%)**	**Incidence rate ratio (95% CI)**	**p value**
**Total physical activity (count per minute)**			
Persistently low	1964 (79·0%)	1·000 (ref)	..
Persistently high	522 (21·0%)	0·934 (0·823–1·060)	0·2914
**Sedentary behaviour**			
Persistently low	480 (19·3%)	1·000 (ref)	..
Persistently average	1472 (59·2%)	1·249 (1·078–1·446)	0·0034
Persistently high	534 (21·5%)	1·282 (1·061–1·548)	0·0100
**Light activity**			
Persistently low	1143 (46·0%)	1·000 (ref)	..
Persistently average	1114 (44·8%)	0·936 (0·843–1·040)	0·2251
Persistently high	229 (9·2%)	0·804 (0·652–0·990)	0·0410
**Moderate-to-vigorous activity**			
Persistently low	1837 (73·9%)	1·000 (ref)	..
Persistently above average	564 (22·7%)	1·006 (0·884–1·145)	0·9286
Persistently high	85 (3·4%)	0·699 (0·515–0·950)	0·0224

Our multiple imputation models included complete data from 4257 participants who had a CIS-R score at age 18 years. We observed similar patterns in our imputed models as in our complete cases analysis, without any substantial differences (appendix p 12), suggesting that our findings are unlikely to be biased by missing data.

In sensitivity analyses, no interactions between physical activity or sedentary behaviour and sex were found, and there were no substantive differences between the findings of our main analyses and any sensitivity analysis (appendix pp 12–13).

## Discussion

To the best of our knowledge, this study is the first prospective study using repeated objective measures to examine associations between physical activity, sedentary behaviour, and depressive symptoms in adolescents. Sedentary behaviour increased and light activity decreased throughout adolescence, and they were consistently associated with depressive symptoms at age 18 years. An additional hour of sedentary behaviour per day was associated with an 8–11% increase in depression score at 18 years, and participants with persistently high or average sedentary behaviour levels between ages 12 years and 16 years had significantly higher depression scores at 18 years compared with those with persistently low sedentary behaviour. An additional hour of light activity per day between age 12 years and 16 years was associated with an 8–11% decrease in depression score, and maintaining persistently high levels of light activity was associated with lower depression scores. Moderate-to-vigorous physical activity only at age 12 years and total physical activity only at ages 12 years and 14 years were associated with reduced depressive symptoms. However, those with persistently high moderate-to-vigorous activity had lower depression scores than those with persistently low moderate-to-vigorous activity. These findings were robust to a series of sensitivity analyses, including models with imputed missing data.

Our data suggest that overall physical activity levels decline and support evidence from other accelerometry studies that sedentary behaviour proportionally displaces light activity throughout adolescence.^24^, ^25^, ^26^ There is a paucity of high-quality, longitudinal research using objective measures to assess the possible harms of rising sedentary behaviour in young people.^14^, ^15^, ^16^, ^27^, ^28^ We have shown, using robust methods, that this rise could increase the risk of adolescent depression, which partially aligns with some cross-sectional accelerometry data in older adults.^29^, ^31^ Data on light activity are also scarce because of the difficulty of recording it without objective measures.^21^ Our results showed that light activity was consistently associated with a reduction in depressive symptoms in adolescents, supporting cross-sectional accelerometry findings in adults.^30^, ^31^

Despite being the focus of most previous research, total physical activity and moderate-to-vigorous physical activity were not consistently associated with depressive symptoms in our study. This finding contrasts with some previous self-report data in adolescents,^8^, ^10^, ^11^, ^12^ but supports findings from the only other accelerometry study in adolescents.^13^ The lack of association between moderate-to-vigorous activity and depressive symptoms at later stages of the study might be related to the fact that activity levels declined throughout the study.

Physical activity might influence depressive symptoms through a variety of psychosocial and biological mechanisms, such as stimulating neuroplasticity in brain regions implicated in depression, reducing inflammation, or promoting self-esteem.^5^ Most studies have shown these effects with moderate-to-vigorous physical activity, but light activity might act through similar pathways. Long periods of sedentary behaviour might negate these protective benefits, potentially increasing the risk of depressive symptoms.

Strengths of this study include its prospective design over 6 years of follow-up, large population-based sample, repeated accelerometry measurement, and rich data on covariates for model adjustments and sensitivity analyses.

The study also had several limitations. Selection bias due to attrition over time or missing data could have influenced the results of our complete case analysis. Although our results did not differ in our fully imputed samples, we only imputed missing data from our subsample (n=4257). The initial ALSPAC sample of children who were alive at 12 months (n=14 901) could have differed from our subsample in ways that affected our results. For example, participants in the full ALSPAC sample were more likely to have higher BMI and less likely to have parents with higher education. There might also have been residual or unmeasured confounding factors that we have not accounted for, such as season or day of accelerometry recording, screen time, physical health, social support, or living environment. It is also possible that our sensitivity analyses include spurious findings,^44^ but we have interpreted our main findings without relying on these results.

The study might have lacked the statistical power to detect consistent associations between moderate-to-vigorous physical activity and depressive symptoms. At baseline, only 1·5% of participants (n=50) were meeting the UK national guidelines of 60 min of moderate-to-vigorous physical activity per day for young people.^45^ However, the 3% of participants identified by our models as having relatively high moderate-to-vigorous activity persistently from age 12 years through 16 years had significantly lower depression scores than the 74% with persistently low moderate-to-vigorous activity. Statistical power was also affected by the sample size decreasing throughout the study, which might partially explain why the p values in our adjusted models were smaller at 12 years than at 14 years and 16 years of age for all exposure variables. Some of these p values might also have been a result of chance due to multiple testing.

Group-based trajectory modelling is useful for identifying distinct developmental trajectories, but relies on polynomial curves that lack flexibility, thus limiting their ability to capture real-world individual variability. Because group assignment is based on the highest probability, some participants with low probabilities for all groups might have been assigned a group that does not fit their individual trajectory well. However, the effect of this is likely to be small, as the average probability values for all groups were at least 0*·7*, and in some groups 0*·9* or higher. Importantly, these are approximations of a continuous reality, and we cannot estimate whether latent groups actually exist in the data.^43^

We also used negative binomial regression to best fit our data, which is commonly applied to count variables. Our outcome variable (CIS-R score) has a similar structure to count data (ie, discrete, independent, and without negative values) and has been used in this way before.^46^ Although CIS-R scores have a severity weighting that is inconsistent with a true count variable, we do not expect our results to have been substantially affected by this difference, as findings from the sensitivity analyses using logistic regression were consistent with our main analysis. Finally, some limitations of using accelerometers include their difficulty in accurately recording certain activities, such as cycling or weightlifting, and inability to record posture, which requires an inclinometer. Furthermore, despite extensive validation,^22^ wearing the accelerometer might have influenced normal physical activity behaviour during the observation period. Accelerometers are also unable to detect contextual information about different types of activity. Findings suggest that mentally active sedentary behaviours, such as working at a desk, are associated with a lower risk of depressive symptoms than mentally passive behaviours, such as watching television.^47^ The concurrent use of objective and self-report measures could be a novel way to examine these potential effect modifiers in greater detail.^48^

Novel approaches are needed to address the global burden of depression. Adolescence represents an important window for intervention given the early onset of depression.^3^, ^4^ Our findings suggest that the displacement of light activity by sedentary behaviour during adolescence could be an important target for public health interventions to reduce the risk of depression. Physical activity guidelines and strategies to promote activity should more directly focus on the mental health benefits of activity, develop specific targets for reducing sedentary behaviour in young people, and promote light activity interventions.

By emphasising the mental health benefits of physical activity, public health bodies could improve adherence to physical activity guidelines by young people, which is currently low.^49^ Physical activity guidelines at present focus on the long-term physical health and developmental benefits of activity for young people, such as improving coordination skills, strengthening bones, or improving cardiovascular fitness.^45^, ^50^ Given the early onset and increasing prevalence of depression in adolescents,^2^, ^3^, ^4^ promoting activity for its mental health benefits could be a more relatable message for young people to increase their activity.

Insufficient high-quality evidence regarding dose–response is available to recommend specific targets for reducing sedentary behaviour in physical activity guidelines,^45^ and developing these recommendations on the basis of self-report data alone could be misleading given the extent to which these studies underestimate daily sedentary behaviour time.^51^ Our data suggested that a 2 h reduction in daily sedentary behaviour between the ages of 12 years and 16 years was associated with a 16–22% reduction in depression scores by age 18 years. For young people with subclinical depressive symptoms, a reduction of this magnitude can have a substantial impact. Specific targets would set measurable and achievable goals that send a stronger public health message for addressing high levels of sedentary behaviour in young people.

Interventions focused around light activity could be a practical method of reducing sedentary behaviour and risk of depression in young people. Current physical activity guidelines primarily discuss light activity as a means of promoting activity in older adults or those with low fitness,^45^, ^50^ probably because of the paucity of studies using objective measures to appropriately investigate light activity. Public health bodies should promote changes at the individual, school, or community levels to incorporate extended bouts of light activity into the daily routines of young people, such as standing lessons, increasing active travel time between classes, or promoting lightly active hobbies such as playing an instrument and painting.

We only investigated depressive symptoms in this study. Future research should use repeated, objective-measure assessments to examine how physical activity and sedentary behaviour are associated with the risk of other mental health conditions, such as anxiety disorders. Future research should also focus on the possible effects of other activity changes in young people, such as the extent to which breaks in sedentary behaviour or meeting national moderate-to-vigorous activity guidelines influence the association between sedentary behaviour and depressive symptoms.

In summary, using objective measures, we found that declining light activity and increasing sedentary behaviour between the ages of 12 years and 16 years were associated with greater depressive symptoms at age 18 years. The displacement of sedentary behaviour with light activity in young people warrants more direct and specific consideration in physical activity guidelines and public health interventions aimed at reducing the prevalence of depression.
