# Supplementary material for: Depressive symptoms and objectively measured physical activity and sedentary behaviour throughout adolescence: a prospective cohort study
Source: Lancet Psychiatry. 2020 Mar;7(3):262–71. doi: 10.1016/S2215-0366(20)30034-1 (PMC7033559; doi:10.1016/S2215-0366(20)30034-1)
Supplement: Supplementary appendix [file mmc1.pdf]

THE LANCET
Psychiatry

Supplementary appendix

This appendix formed part of the original submission and has been peer reviewed. We post it as supplied by the authors.

Supplement to: Kandola A, Lewis G, Osborn DPJ, Stubbs B, Hayes JF, et al. Depressive symptoms and objectively measured physical activity and sedentary behaviour throughout adolescence: a prospective cohort study. *Lancet Psychiatry* 2020; published online February 11. http://dx.doi.org/10.1016/S2215-0366(20)30034-1.

Appendix

Figure 1. Flow chart of participants

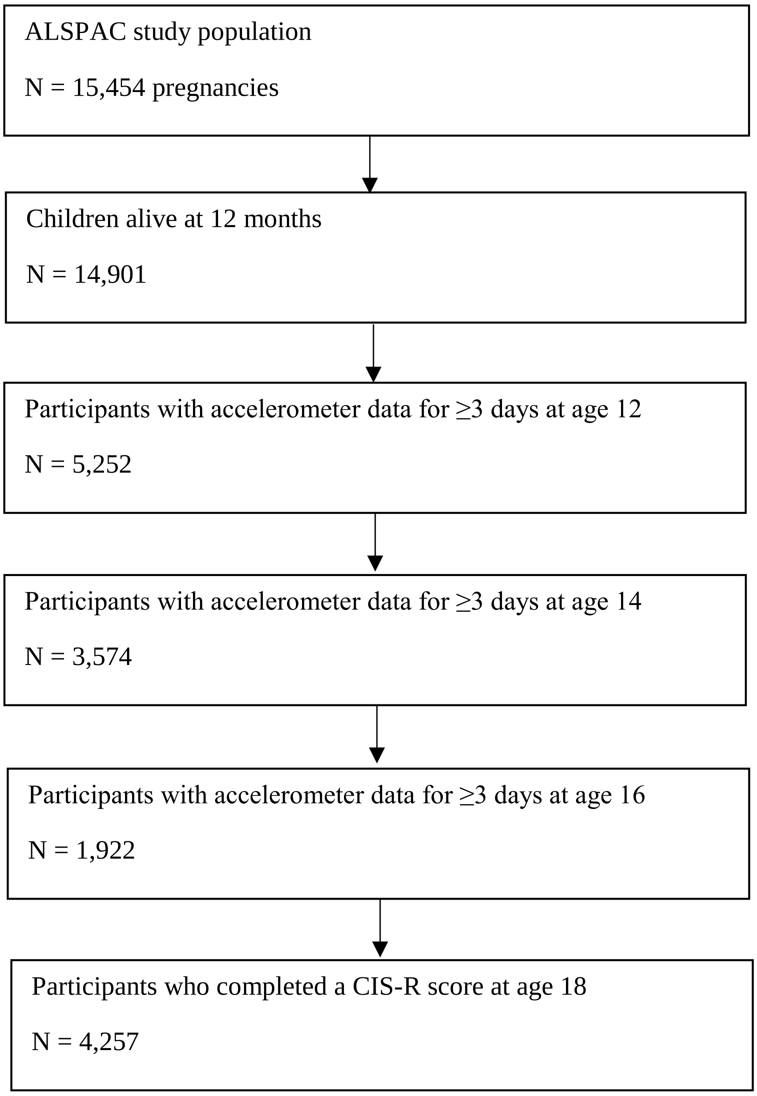

ALSPAC = Avon Longitudinal Study of Parents and Children.

Figure 2. PA, depression, and covariates Directed Acyclic Graph (DAG)

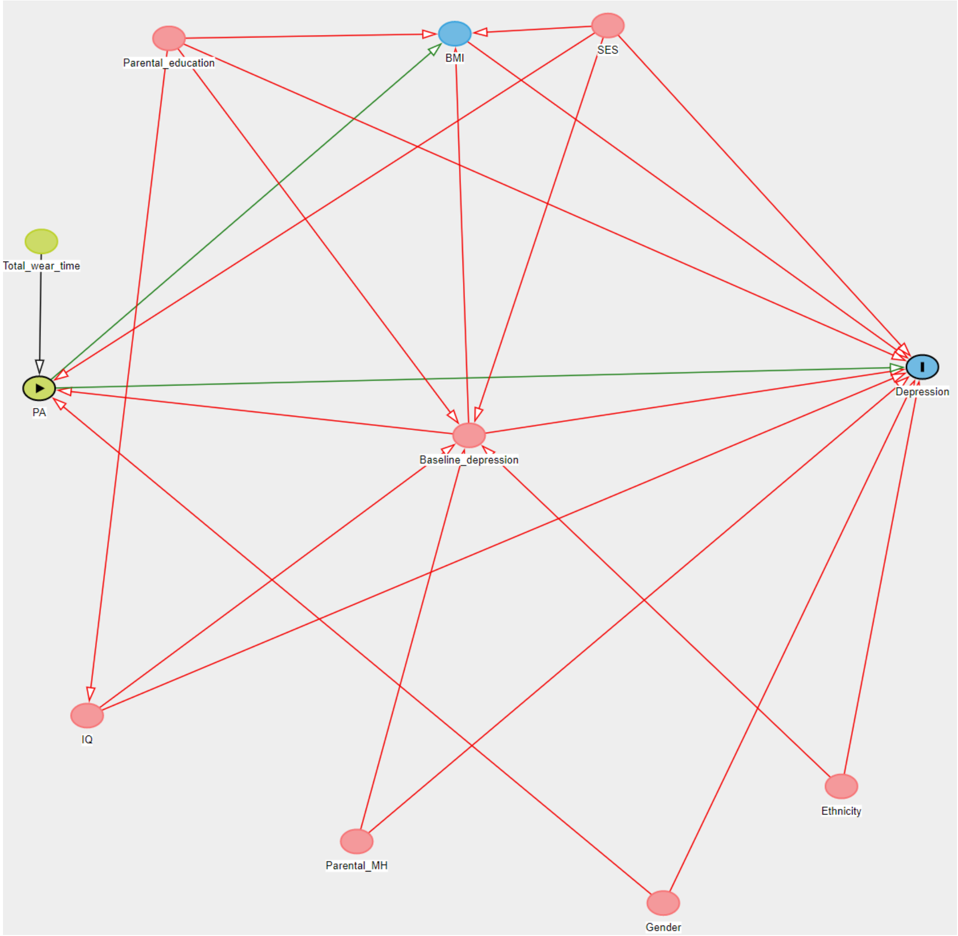

The following DAG was made using the online resource DAGitty (http://www.dagitty.net/dags.html#). The variables and their relationships included in the DAG are the product of discussions between co-authors, leading to several iterations before the final model. We only include variables in the DAG that were available to us at baseline.

Methods 1. Group based trajectory modelling

Group based trajectory modelling is a form of finite mixture modelling that can identify unobserved (latent) subgroups of people with statistically similar PA trajectories (1). The method uses a finite set of polynomial functions of age or time to describe the trajectory for each group, based on maximum likelihood estimation. After estimating groups and their trajectories, each participant is assigned a probability of group membership and allocated to the group they have the highest probability.

We conducted our Group based trajectory modelling using the traj command in STATA. We specified the final number of groups and their polynomial functions based on their Bayesian Information Criterion (BIC), an adequate sample size in each group, an average posterior probability (APP) value of >= 0.7, and the interpretability of the model for explaining the data (2). The model imputes missing values using maximum likelihood estimates (3).

Using the trajplot command in STATA, we generated the following figures to illustrate the trajectory subgroups used in our models. We created Group based trajectory models for total PA (CPM), sedentary, light activity and MVPA time, and baseline depression (MFQ scores).

Figure 3. CIS-R distribution

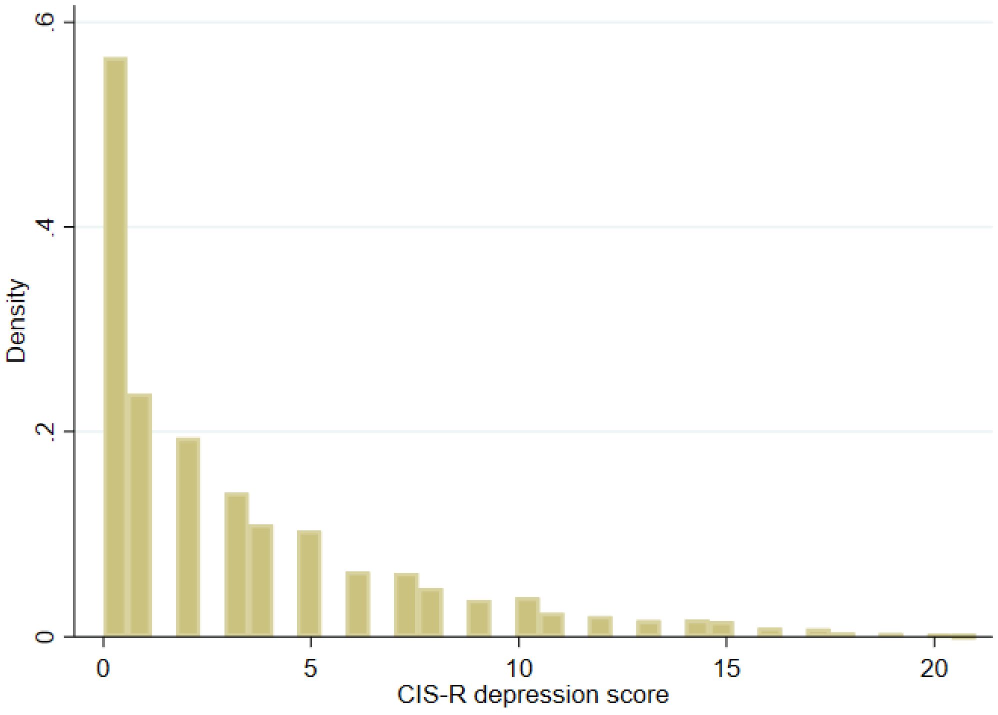

Figure 4. Total PA (CPM) trajectories

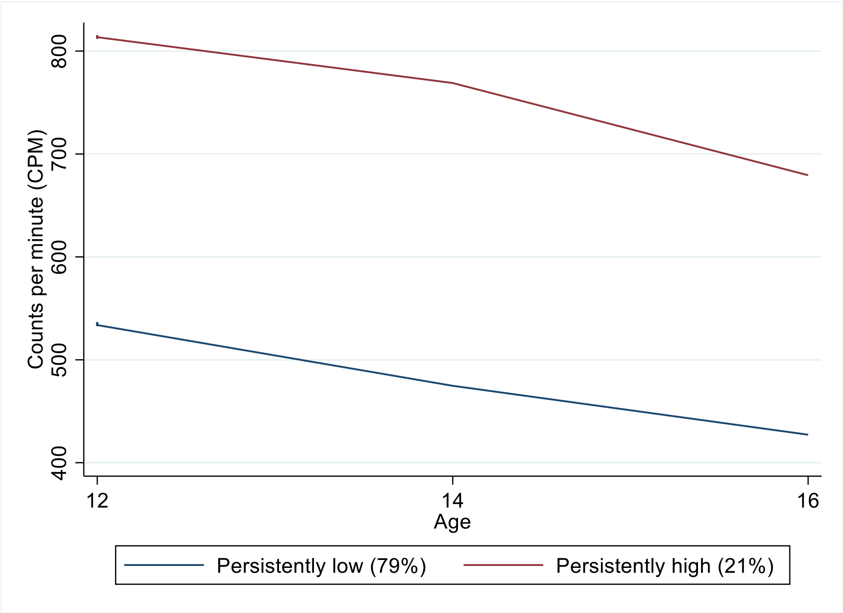

Figure 5. Sedentary time trajectories

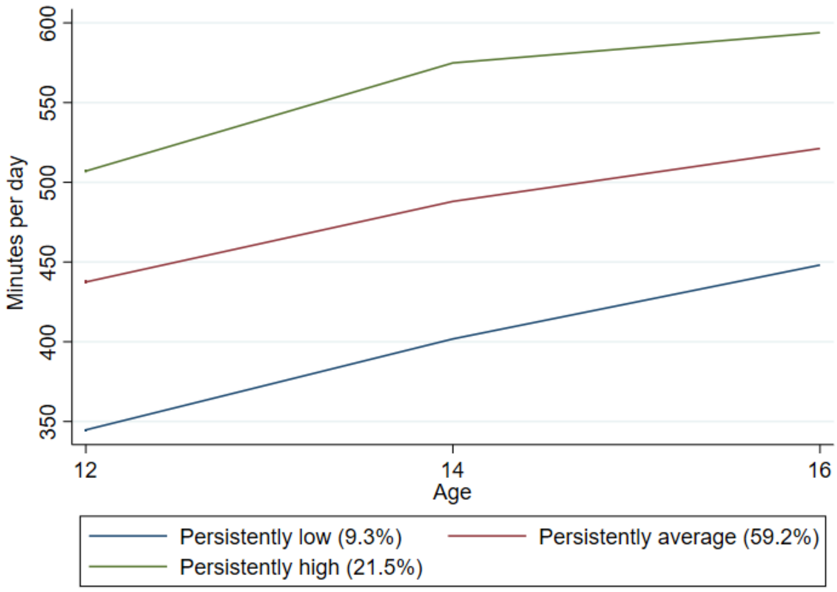

Figure 6. Light time trajectories

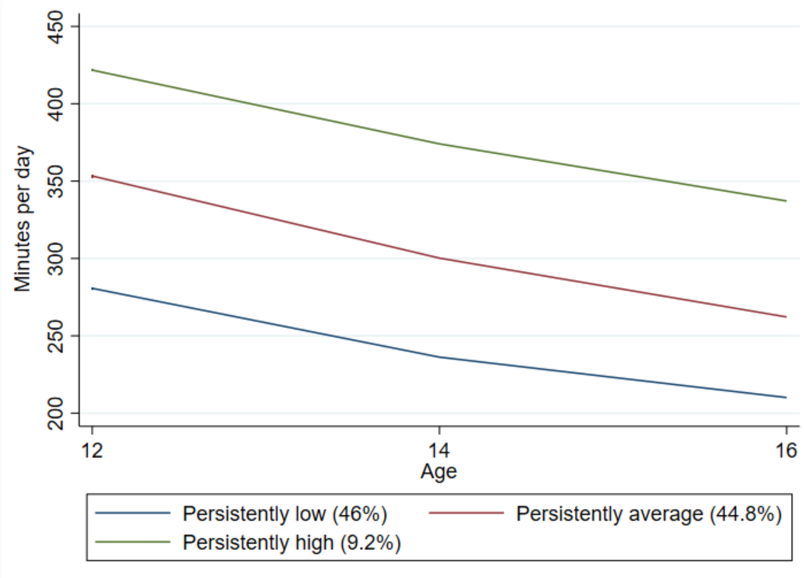

Figure 7. MVPA time trajectories

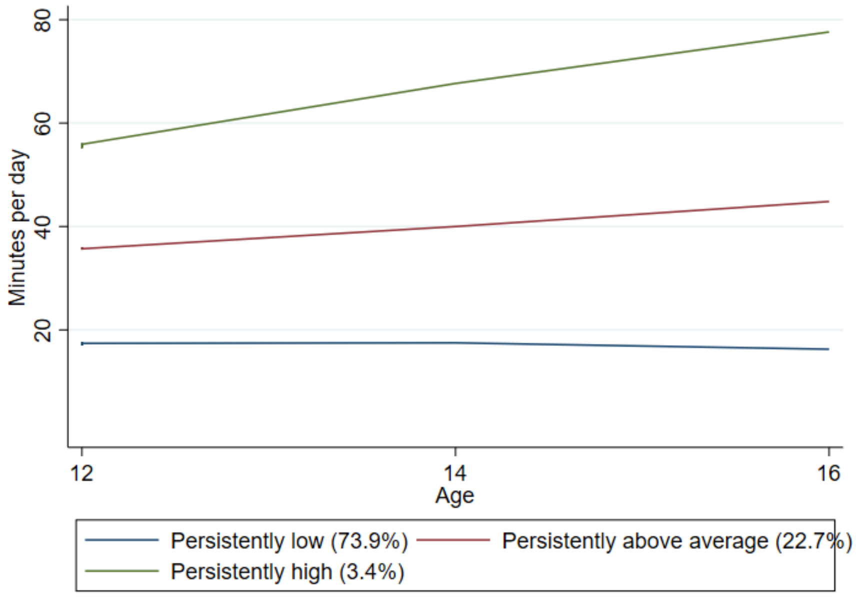

Figure 8. Baseline depression trajectories (MFQ)

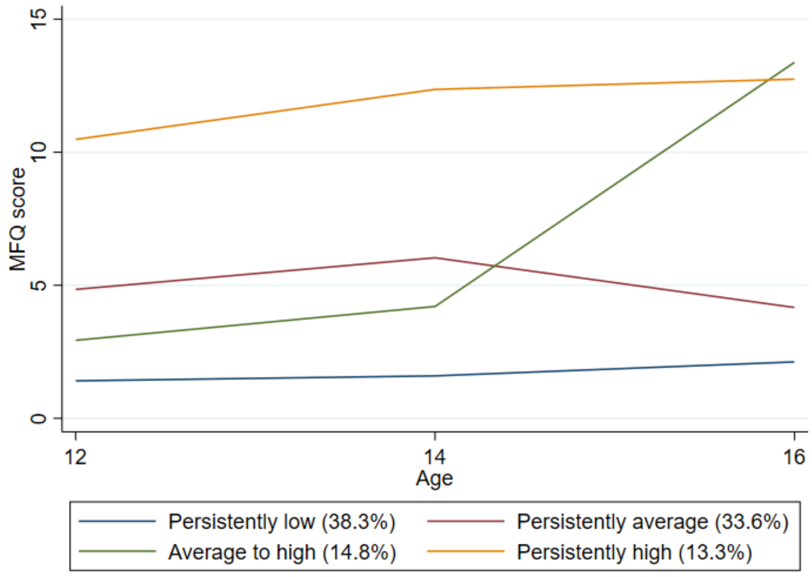

Table 1. Baseline characteristics of included and excluded participants

**Baseline characteristic**		**Incidence/total no. of participants with available data (%)**		***P***
		**Included (n = 4,257)**	**Excluded (n = 10,664)**	
**Sex**	Female	2,390/4,257 (56.14)	4,351/10,664 (40.80)	<0.001
**Ethnicity**	Non-white	173/4062 (4.26)	429/7,912 (5.42)	0.006
**Parental education**	Higher (degree)	1,207/4,257 (28.35)	1,328/10,664 (12.45)	<0.001
**Maternal social class**	Manual	553/3626 (15.25)	1,435/6,354 (22.58)	<0.001
**Parental psychiatric diagnosis**	Severe depression or schizophrenia	422/4257 (9.91)	1,117/10,664 (10.47)	0.065
**BMI**	Overweight or obese	270/3671 (7.35)	263/2602 (10.11)	<0.001

**Baseline depression**	MFQ > = 10	344/3,683 (9.34)	227/2600 (8.73)	0.408

OR: Odds ratio; CIs: Confidence intervals

Table 2. Physical activity at ages 12, 14, and 16

Age (years)	Physical activity variable	Boys		Girls		All	
		Mean or median (SD or IQR 25-75)	% of total wear time	Mean or median (SD or IQR 25-75)	% of total wear time	Mean or median (SD or IQR 25-75)	% of total wear time
**12**	Total activity (CPM)	662.04 (184.92)	n/a	547.97 (151.51)	n/a	603.33 (177.62)	n/a
	Sedentary activity minutes	421.61 (66.13)	53.74	439.53 (64.35)	56.66	430.99 (65.80)	55.26
	Light activity minutes	334.28 (59.09)	42.59	317.82 (56.02)	41.01	325.66 (58.09)	41.76
	MVPA activity minutes	25.67 (16.29, 38.67)	3.25	15.59 (9.67, 24.29)	2.01	20 (11.86, 31.21)	2.55
**14**	Total activity (CPM)	598.61 (192.38)	n/a	486.67 (153.15)	n/a	539.12 (181.43)	n/a
	Sedentary activity minutes	471.35 (70.16)	59.40	500.22 (63.66)	63.62	486.69 (68.30)	61.64
	Light activity minutes	293.04 (59.10)	36.94	265.93 (51.30)	33.85	278.63 (56.72)	35.29
	MVPA activity minutes	25.82 (15.34, 38.69)	3.29	16.8 (9.67, 27)	2.15	20.71 (12, 32.8)	2.63
**16**	Total activity (CPM)	529.90 (166.35)	n/a	430.60 (137.16)	n/a	474.83 (158.68)	n/a
	Sedentary activity minutes	515.60 (68.27)	64.43	528.99 (62.13)	67.51	523.02 (65.25)	66.14
	Light activity minutes	255.67 (58.32)	31.90	236.31 (50.76)	30.13	244.94 (55.08)	30.92
	MVPA activity minutes	25.69 (16.8, 39.86)	3.22	15 (7.17, 25.71)	1.90	19.5 (10.25, 33.34)	2.47

SD: Standard deviation; IQR: Interquartile range; CPM: Counts per minute

Table 3. Model iterations for group-based trajectory models, according to number of groups and trajectory shapes

Variable	Number of groups	Trajectory shape (0 = zero order, 1 = linear, 2 = quadratic)	BIC for total number of observations (N = 3519)	% group membership				
				1	2	3	4	5
**CPM**	2	0 0	-46321.36	79.48	20.52			
	2	0 1	-46244.02	73.60	26.40			
	2	0 2	-46244.53	78.99	21.01			

	2	1 1	-46027.21	73.79	26.21			
	2	1 2	-46027.33	79.04	20.96			
	2	2 2	-46029.34	78.98	21.02			
	3	0 0 0	-46246.71	66.10	29.53	4.37		
	3	0 1 1	-46227.35	67.21	30.01	2.78		
	3	0 1 2	-46225.30	62.04	31.45	6.51		
	3	0 2 2	-46211.46	66.85	33.15	2.36		
	3	1 1 1	-46111.89	67.53	29.26	2.61		
	3	1 1 2	-46109.52	67.88	28.94	3.18		
	3	1 2 1	-46104.72	69.33	25.68	4.99		
	3	1 2 2	-46107.39	70.45	25.62	3.93		
	3	2 1 1	-46189.43	65.69	28.65	5.66		
	3	2 2 1	-46080.71	75.56	19.35	5.09		
	3	2 1 2	-46071.41	70.58	21.12	8.3		
	3	2 2 1	-46246.71	66.65	30.22	3.13		
**Sedentary**	2	0 0	-40580.56	32.34	67.64			
	2	0 1	-39663.92	11.47	88.53			

	2	0 2	-39639.21	12.23	87.76			
	2	1 1	-39478.15	36.95	63.05			
	2	1 2	-39451.10	37.15	62.87			
	2	2 2	-39453.79	37.41	62.59			
	3	0 0 0	-40581.83	20.99	71.43	7.57		
	3	0 1 1	-39477.44	1.46	41.63	56.90		
	3	0 1 2	-39449.30	1.54	41.84	56.60		
	3	0 2 2	-39451.10	1.68	42.65	55.65		
	3	1 1 1	-39438.30	20.64	60.47	18.89		
	3	1 1 2	-39414.33	16.29	54.21	29.48		
	3	1 2 1	-39418.11	24.89	63.89	11.22		
	3	1 2 2	-39410.23	19.26	59.23	21.50		
	3	2 1 0	-39470.96	34.25	64.59	1.14		
	3	2 1 1	-39439.66	21.53	60.64	17.82		
	3	2 1 2	-39416.15	17.02	54.36	28.61		
	3	2 2 1	-39421.50	25.13	63.94	10.91		
	3	2 2 2	-39413.45	19.52	59.24	21.22		

	4	0 0 0 0	-40589.08	2.56	27.29	66.18	3.94	
**Light**	2	0 0	-39499.75	77.69	22.30			
	2	0 1	-38643.13	8.59	91.40			
	2	0 2	-38633.93	8.30	91.69			
	2	1 1	-38272.04	69.78	30.21			
	2	1 2	-38271.05	69.18	30.81			
	2	2 2	-38258.23	69.64	30.35			
	3	0 0 0	-39499.39	68.25	29.66	2.09		
	3	1 2 1	-38196.20	43.53	46.27	10.18		
	3	2 1 1	-38256.63	7.51	66.73	25.74		
	3	2 2 1	-38189.06	46.05	44.78	9.15		
	3	2 2 2	-38182.65	46.01	44.80	9.17		
**MVPA**	2	0 0	-29158.27	83.03	16.96			
	2	0 1	-29111.67	81.84	18.15			
	2	0 2	-29115.15	81.85	18.14			
	2	1 1	-29115.61	81.80	18.19			
	2	1 2	-29119.06	81.81	18.18			

	2	2 2	-29121.40	81.83	18.16			
	3	1 1 1	-28991.31	73.90	22.66	3.42		
	3	1 1 2	-28995.14	74.10	22.61	3.27		
	3	1 2 2	-29007.30	76.67	16.91	6.40		
	3	2 1 0	-29008.14	74.10	21.94	3.95		
	3	2 1 1	-29124.31	25.64	58.14	16.21		
	3	2 1 2	-29007.30	16.91	76.67	6.40		
	3	2 2 1	-29122.26	32.28	52.92	14.79		
	3	2 2 2	-29000.97	74.30	22.50	3.19		
**MFQ**	2	0 1	-44943.13	76.88	23.11			
	2	0 2	-44917.35	77.10	22.89			
	2	1 1	-44836.23	78.54	21.45			
	2	1 2	-44808.87	78.70	21.29			
	2	2 2	-44810.34	78.76	21.23			
	3	0 0 0	-45175.98	63.51	28.90	7.57		
	3	0 1 1	-44611.78	55.92	33.81	10.26		
	3	0 1 2	-44584.65	53.83	35.07	11.08		

	3	0 2 2	-44582.67	54.15	35.03	10.80		
	3	1 1 1	-44552.03	62.24	28.64	9.10		
	3	1 1 2	-44531.05	60.12	29.86	10.01		
	3	1 2 1	-44538.40	63.51	27.85	8.63		
	3	1 2 2	-44536.99	60.95	29.49	9.55		
	3	2 1 0	-44594.72	70.65	10.98	18.35		
	3	2 1 1	-44553.37	62.54	28.40	9.04		
	3	2 1 2	-44532.65	60.47	29.58	9.93		
	3	2 2 1	-44541.50	63.61	27.78	8.60		
	3	2 2 2	-44529.85	61.11	29.35	9.52		
	3	1 2 3	-44528.25	60.69	29.53	9.77		
	4	0 0 0 0	-46198.98	22.13	39.82	27.55	10.47	
	4	0 1 1 1	-44905.80	34.39	27.02	16.80	21.77	
	4	1 1 1 1	-44727.95	36.54	20.35	15.92	27.17	
	4	1 1 1 2	-44618.05	21.10	36.80	25.90	16.19	
	4	1 1 2 2	-44551.40	21.27	37.17	26.17	15.37	
	4	1 2 2 2	-44426.99	38.51	33.40	14.92	13.33	

	4	2 2 2 2	-44501.21	20.41	36.98	27.17	15.42	
	5	0 0 0 0 0	-48793.35	20.24	17.50	36.77	10.41	15.05
	5	1 1 1 1 1	-48568.81	20.11	16.82	36.30	14.79	11.96
	5	1 2 2 2 2	-482715.69	17.00	20.37	37.07	15.10	10.44
	5	2 2 2 2 2	-47715.18	37.06	16.67	10.78	15.32	20.15

BIC: Bayesian Information Criterion; CPM: Counts per minute; MVPA: Moderate to vigorous physical activity; MFQ: Moods and Feelings Questionnaire.

Methods 2. Multiple imputation models

We conducted multiple imputations with chained equations to create "complete" 30 datasets (4). Missing values are imputed based on observed values for a given individual. The imputed dataset contains complete data from 4,257 participants, which is the total number of participants with a complete CIS-R depression score at age 18. Results from each of the 30 imputed dataset were pooled into a single multiple-imputation result. Multiple imputation yields accurate standard errors and take account of the uncertainty of the predictions because multiple predictions are made for each missing value.

Our multiple imputation model contained all exposure, outcome and confounding variables: CIS-R depression score at 18, total CPM, sedentary, light, MVPA time at ages 12, 14, and 16, maternal social class, ethnicity, mental health diagnoses at age 13, BMI at ages 11, 13 and 17, MFQ at ages 12, 14, and 16, gender, parental mental health, parental education, and IQ at age 8. We included three further variables to improve prediction of missingness in the ALSPAC cohort: maternal smoking during pregnancy, alcohol use at age 13, and psychosis (at least one reported episode of psychosis) at age 16. For non-normally distributed variables, we use predictive mean matching.

Table 4. Cross-sectional and longitudinal associations between PA and depression scores from imputed sample (n = 4,257)

	Age	CPM (per 100)			Time spent at different intensities								
					Sedentary (per 60 mins)			Light (per 60 mins)			MVPA (per 15 mins)		
		IRR	95% CI	*P*	IRR	95% CI	*P*	IRR	95% CI	*P*	IRR	95% CI	*P*
**Cross-sectional**	12	0.966	0.948, 0.984	<0.001	1.038	1.007, 1.068	0.014	0.947	0.917, 0.979	0.001	0.947	0.917, 1.021	0.001
**Longitudinal**	12	0.950	0.925, 0.976	<0.001	1.048	1.008, 1.091	0.020	0.946	0.990, 0.904	0.016	0.915	0.873, 0.960	<0.001
	14	0.964	0.939, 0.989	0.006	1.083	1.040, 1.129	<0.001	0.957	0.906, 1.011	0.114	0.971	0.933, 1.119	0.158
	16	0.973	0.942, 1.005	0.095	1.054	1.001, 1.113	0.046	0.928	0.875, 0.985	0.014	0.839	0.936, 1.030	0.474

All models are fully adjusted for sex, ethnicity, maternal social class, paternal psychiatric history, paternal education,, and baseline depression.

Table 5. Logistic models with depression as a dichotomous outcome

Age (years)	Model (n)	CPM (per 100)			Time spent at different intensities								
					Sedentary (per 60 mins)			Light (per 60 mins)			MVPA (per 15 mins)		
		OR	95% CI	*P*	OR	95% CI	*P*	OR	95% CI	*P*	OR	95% CI	*P*
**12**	Fully adjusted (2,486)	0.93	0.87, 0.99	0.049	1.15	1.02, 1.30	0.022	0.83	0.77, - 0.99	0.039	0.89	0.78, 1.01	0.078
**14**	Fully adjusted (1,938)	0.91	0.84, 0.98	0.017	1.24	1.08, 1.42	0.002	0.80	0.69, - 0.93	0.004	0.90	0.79, 1.02	0.108
**16**	Fully adjusted (1,220)	1.02	0.91, 1.14	0.771	1.10	0.90, 1.33	0.352	0.88	0.71, - 1.08	0.225	1.05	0.91, 1.22	0.505

Table 6. Longitudinal models excluding participants with elevated depressive symptoms (MFQ >= 10)

Age (years)	Model (n)	CPM (per 100)			Time spent at different intensities								
					Sedentary (per 60 mins)			Light (per 60 mins)			MVPA (per 15 mins)		
		IRR	95% CI	*P*	IRR	95% CI	*P*	IRR	95% CI	*P*	IRR	95% CI	*P*
**12**	Fully adjusted (2,163)	0.937	0.904, 0.970	<0.001	1.075	1.017, 1.136	0.011	0.904	0.850, 0.961	0.001	0.900	0.844, 0.959	0.001
**14**	Fully adjusted (1,760)	0.960	0.925, 0.997	0.034	1.080	1.017, 1.180	0.012	0.931	0.864, 1.004	0.064	0.964	0.906, 1.026	0.251
**16**	Fully adjusted (1,062)	0.972	0.922, 1.024	0.286	1.087	1.008, 1.172	0.031	0.905	0.825, 0.993	0.034	0.992	0.924, 1.065	0.829

Table 7. Longitudinal models including BMI

Age (years)	Model (n)	CPM (per 100)			Time spent at different intensities								
					Sedentary (per 60 mins)			Light (per 60 mins)			MVPA (per 15 mins)		
		IRR	95% CI	*P*	IRR	95% CI	*P*	IRR	95% CI	*P*	IRR	95% CI	*P*
**12**	Fully adjusted (2,348)	0.946	0.916, 0.978	0.001	1.070	1.017, 1.127	0.010	0.923	0.871, 0.977	0.006	0.904	0.848, 0.964	0.002
**14**	Fully adjusted (1,916)	0.966	0.933, 1.001	0.056	1.072	1.014, 1.133	0.014	0.952	0.890, 1.019	0.159	0.966	0.911, 1.023	0.241
**16**	Fully adjusted (1,151)	0.985	0.938, 1.034	0.548	1.077	1.004, 1.116	0.038	0.919	0.844, 1.001	0.053	1.005	0.942, 1.073	0.874

Table 8. Longitudinal models excluding participants with smoking at 16 and alcohol use at 15 as confounding variable

Age (years)	Model (n)	CPM (per 100)			Time spent at different intensities								
					Sedentary (per 60 mins)			Light (per 60 mins)			MVPA (per 15 mins)		
		IRR	95% CI	P	IRR	95% CI	P	IRR	95% CI	P	IRR	95% CI	P
**12**	Fully adjusted (1,799)	0.955	0.920, 0.992	0.019	1.050	0.991, 1.113	0.101	0.926	0.868, 0.988	0.021	0.908	0.843, 0.977	0.010
**14**	Fully adjusted (1,536)	0.970	0.933, 1.008	0.128	1.076	1.011, 1.145	0.021	0.955	0.885, 1.030	0.237	0.970	0.909, 1.003	0.348
**16**	Fully adjusted (1,019)	0.984	0.932, 1.038	0.549	1.043	0.968, 1.124	0.269	0.920	0.840, 1.001	0.069	0.990	0.922, 1.062	0.777

**Table 9. STROBE Statement**	**Item No**	**Recommendation**	**Reported on manuscript page**
**Title and abstract**	1	(*a*) Indicate the study's design with a commonly used term in the title or the abstract	2
		(*b*) Provide in the abstract an informative and balanced summary of what was done and what was found	2
**Introduction**			
Background/rationale	2	Explain the scientific background and rationale for the investigation being reported	4-5
Objectives	3	State specific objectives, including any prespecified hypotheses	5
**Methods**			
Study design	4	Present key elements of study design early in the paper	5-9
Setting	5	Describe the setting, locations, and relevant dates, including periods of recruitment, exposure, follow-up, and data collection	5-7
Participants	6	(*a*) Give the eligibility criteria, and the sources and methods of selection of participants. Describe methods of follow-up	5
		(*b*) For matched studies, give matching criteria and number of exposed and unexposed	-
Variables	7	Clearly define all outcomes, exposures, predictors, potential confounders, and effect modifiers. Give diagnostic criteria, if applicable	6-7
Data sources/ measurement	8*	For each variable of interest, give sources of data and details of methods of assessment (measurement). Describe comparability of assessment methods if there is more than one group	6-7
Bias	9	Describe any efforts to address potential sources of bias	7-9
Study size	10	Explain how the study size was arrived at	
Quantitative variables	11	Explain how quantitative variables were handled in the analyses. If applicable, describe which groupings were chosen and why	7-9
Statistical methods	12	(*a*) Describe all statistical methods, including those used to control for confounding	7-9
		(*b*) Describe any methods used to examine subgroups and interactions	7-9
		(*c*) Explain how missing data were addressed	9
		(*d*) If applicable, explain how loss to follow-up was addressed	9
		(*e*) Describe any sensitivity analyses	8-9
**Results**			
Participants	13*	(a) Report numbers of individuals at each stage of study—eg numbers potentially eligible, examined for eligibility, confirmed eligible, included in the study, completing follow-up, and analysed	9
		(b) Give reasons for non-participation at each stage	9

		(c) Consider use of a flow diagram	Supplementary page 1 (Figure1)
Descriptive data	14*	(a) Give characteristics of study participants (eg demographic, clinical, social) and information on exposures and potential confounders	9-10 and supplementary page 5, Figure 2
		(b) Indicate number of participants with missing data for each variable of interest	9-10
		(c) Summarise follow-up time (eg, average and total amount)	9
Outcome data	15*	Report numbers of outcome events or summary measures over time	11
Main results	16	(*a*) Give unadjusted estimates and, if applicable, confounder-adjusted estimates and their precision (eg, 95% confidence interval). Make clear which confounders were adjusted for and why they were included	12
		(*b*) Report category boundaries when continuous variables were categorized	12-13
		(*c*) If relevant, consider translating estimates of relative risk into absolute risk for a meaningful time period	-
Other analyses	17	Report other analyses done—eg analyses of subgroups and interactions, and sensitivity analyses	13-14<br>And supplementary pages 3-9
**Discussion**			
Key results	18	Summarise key results with reference to study objectives	14
Limitations	19	Discuss limitations of the study, taking into account sources of potential bias or imprecision. Discuss both direction and magnitude of any potential bias	15-16
Interpretation	20	Give a cautious overall interpretation of results considering objectives, limitations, multiplicity of analyses, results from similar studies, and other relevant evidence	14-17
Generalisability	21	Discuss the generalisability (external validity) of the study results	14-17
**Other information**			
Funding	22	Give the source of funding and the role of the funders for the present study and, if applicable, for the original study on which the present article is based	3

References

1. Nagin D, Tremblay RE. Trajectories of Boys' Physical Aggression, Opposition, and Hyperactivity on the Path to Physically Violent and Nonviolent Juvenile Delinquency. Child Dev [Internet]. 1999 Sep 1;70(5):1181–96.

2. Nagin DS, Odgers CL. Group-Based Trajectory Modeling in Clinical Research. Annu Rev Clin Psychol [Internet]. 2010 Mar;6(1):109–38.

3. Nagin D. Group-based modeling of development. Harvard University Press; 2005. 201 p.

4. White IR, Royston P, Wood AM. Multiple imputation using chained equations: Issues and guidance for practice. Stat Med. 2011 Feb 20;30(4):377–99.
